# Medical respite post-hospitalization for adults experiencing homelessness

**DOI:** 10.1097/01.NURSE.0000918524.41501.98

**Published:** 2023-02-23

**Authors:** Cindy Hadenfeldt, Martha J. Todd, Chelsea Hamzhie

**Affiliations:** At the Creighton University College of Nursing, **Cindy Hadenfeldt** is an associate professor, **Martha Todd** is a professor, and **Chelsea Hamzhie** is an assistant professor. The authors acknowledge the collaborative support of Creighton University's Kingfisher Arts program and the Health and Housing Coalition and Medical Respite Steering Committee.

**Keywords:** community assessment, homelessness, medical respite

## Abstract

Nurses provide care in various settings and advocate for vulnerable populations. Recognizing the need for follow-up care after hospitalization and mobilizing necessary resources are part of caring for patients, including those experiencing homelessness. This article discusses how one community coalition assessed gaps in care that might be met by establishing medical respite in the community.

## Abstract 2

This article discusses how one community coalition assessed gaps in care that might be met by establishing medical respite in the community.

## Case example

**Figure:**
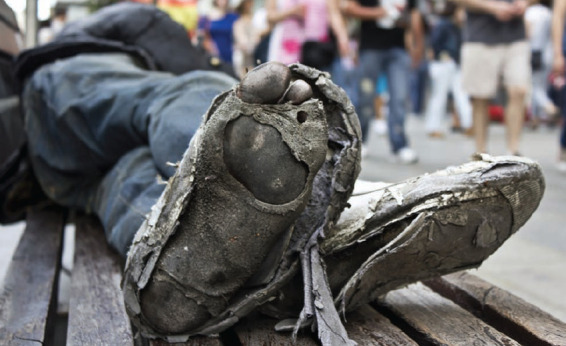
No caption available.

Mary was hospitalized for diabetes and acute bilateral lower extremity cellulitis. Her blood glucose levels were elevated, with an admission A1C of 13%.

Prior to hospitalization, Mary resided in a homeless shelter. After 10 days in the medical-surgical unit receiving I.V. antibiotics, analgesics, and twice-daily sterile dressing changes, the discharge planner could not place Mary in a skilled facility for further care due to a lack of personal finances and health insurance. In addition, family members were unwilling to take her in because they lived out of state and felt incapable of helping with managing her diabetes and performing Mary's frequent requisite dressing changes.

Rather than being dismissed to the street or a homeless shelter ill-equipped to provide for her needs, Mary was discharged to a medical respite facility for adults experiencing homelessness. There, she would receive assistance with medication administration, dressing changes, and learning how to care for herself from nurses and other healthcare professionals. Nursing support in the respite setting while transitioning Mary from the hospital to self-care at home or in a shelter would include helping her achieve glycemic control to prevent the long-term complications of diabetes.

## Introduction

Medical respite care facilities, often called recuperative care, provide “acute and post-acute care for persons experiencing homelessness who are too ill or frail to recover from physical illness or injury on the streets but are not ill enough to be in a hospital.”^1^ Patients at these facilities benefit from an interprofessional approach to providing care and resources.^1^

Approximately 116 medical respite facilities have been established in 38 states, mostly in urban areas with greater homeless populations. However, many geographical regions in the US remain underserved.^2^ Medical respite traditionally provides 1 week to 2 months of daily health monitoring and appropriate-level care in a safe and supportive environment. During this time, patients receive the care necessary for recovery and are connected with essential services, such as case management, disability, and housing.^3^

This article describes how one community coalition of academic nurse consultants assessed community needs when no medical respite care was available. The nurses identified healthcare service gaps for adults with serious health issues post-hospitalization that might be met by establishing medical respite in the community. As a result of this collaboration, a pilot medical respite program was implemented in the community.

### Homelessness

In 2022, the National Alliance to End Homelessness reported that 580,466 people experience homelessness each night in the US.^4^ Sixty-one percent of people experiencing homelessness reside in shelters that provide a place to sleep, meals, and programs to help them receive services to overcome obstacles contributing to their homelessness.^4^ The remaining individuals experiencing homelessness live unsheltered in areas including sidewalks, subway trains, vehicles, and parks.^4^

Health concerns for individuals experiencing homelessness are complex and often go untreated or undertreated, resulting in poor health outcomes. Those unsheltered individuals experience premature mortality, averaging 12 years earlier than their housed counterparts.^5^ Rates of depression and substance use disorders are significantly higher among those experiencing homelessness than the general population. Similarly, rates of diabetes, hypertension, HIV infection, and hepatitis C virus infection follow this trend.^5^ Exposure to communicable diseases, harmful weather extremes, and violence may be greater due to homelessness. It can exacerbate health conditions as well as contribute to new pathology.^5^ The COVID-19 pandemic added a burden to this already vulnerable population.^6^

### Barriers to care

EDs often become the primary source of healthcare for individuals who are homeless due to a lack of insurance and the inability to pay for healthcare services. Persons experiencing homelessness have increased ED visits.^7^ The annual number of ED visits is 42 per 100 persons in the general population, compared with the rate of 203 ED visits per 100 homeless persons.^7^ Therefore, transitional care from ED visits and hospitalizations to independent self-care is vital.

Transportation to follow-up appointments with care providers may not be available following hospitalization, and the individual may rely on public transport, such as city buses. Those who are unemployed are financially limited and may not be able to access or afford medications without health insurance from an employer.

Homeless residential shelters may not be able to provide higher-level healthcare.^8^ Many shelters do not employ nursing personnel. They expect that individuals will be independent in their activities of daily living (ADL) while residing at the shelter and manage any wound dressing changes, medication administration, and ambulation without assistance. Older adults experiencing homelessness may have more chronic illnesses and require assistance that the shelter cannot provide.^9^

### Post-hospitalization health needs

At hospital discharge, adults experiencing homelessness need a respectful and understanding approach to care, housing assessments, communication and coordination, support for after-care, complex medical care and medication management, and basic needs and transportation.^10^

In areas without medical respite care, providers from hospitals and community-based agencies may coordinate the services needed for persons experiencing homelessness for appropriate care and recovery after an ED visit or hospitalization.^11^ Service providers have reported remorse and frustration at the lack of processes and care provided to these individuals after discharge; similarly, individuals experiencing homelessness reported stress and uncertainty about post-hospitalization care.^11^

### Nurses' role

Nurses are integral in coordinating and transitioning care through hospitalization and discharge. In a medical respite facility, nurses determine qualifications for admission and discharge, establish and coordinate care, provide wellness checks, ensure that medications are appropriately administered, monitor for adverse reactions, and teach patients how to care for themselves.

Positive outcomes from recovering in medical respite facilities include reduced hospital admissions, shortened hospital lengths of stay, decreased frequency of ED admissions, and increased use of primary care services.^2^

Complex healthcare needs are common among people experiencing homelessness. Nurses can teach individuals to care for themselves, connect them to resources, and offer support during this process.

## Starting a community coalition

In a community located in the midwest region of the US, no respite care was available, and hospitalized patients who were homeless experienced prolonged lengths of stay due to the inability to discharge promptly to appropriate level facilities. Hospitals were not financially compensated for this extended care; homeless shelter personnel admitted individuals into their facilities without the licensed personnel to provide the requisite levels of care.

The coalition conducted a qualitative study to assess community needs, describe current realities, and identify healthcare service gaps for adults experiencing homelessness. The coalition observed three distinct groups of participants to gain broad perspectives of those impacted by this lack of resources: adults experiencing homelessness, hospital discharge planners, and shelter staff.

## Three perspectives

An Institutional Review Board at a Midwestern private university approved the study. The setting was an urbanized region of 4,346.3 square miles with approximately 972,195 people.^12^

**Table:** Demographic results, group 1 (N = 20)

Age	Range: 29-70 years Mean age: 50 years
Gender	Male: 70% Female: 30%
Race	Non-Hispanic White: 55% Hispanic: 5% Black American: 25% Native American: 15%
Highest level of education completed	Middle school: 15% High school/GED: 50% College education: 35%
Number of times homeless	First time: 35% Multiple times: 65%

The area has eight residential homeless facilities, three federally qualified health centers, and other supportive service agencies to address the homeless population's needs. Descriptive statistics and summarizing key points from the transcripts were used to identify gaps in care.

## Participants

The first group consisted of guests at a homeless shelter who had been hospitalized or received care in an ED the previous year. The shelter facility staff recommended these participants to the investigators as meeting inclusion criteria. Participants were selected after an interview. Before the interview, the investigator presented the purpose of the study as well as the risks and benefits of participating, answered questions, and obtained written informed consent. The shelter staff then introduced each participant to the two RN academic faculty and provided a quiet place for the interview.

A convenience sample of 20 adults participated in semistructured interviews with the investigators. Interviews were recorded with permission and transcribed verbatim. In addition to basic demographic information, participants were asked about their health status, including their ability to perform activities of daily living, such as eating, dressing, toileting, and bathing; their ability to walk; sensory impairment; the presence of pain; history of falls; and problems with depression, anxiety, and other mental health concerns. Participants were also asked to describe their difficulties or supports when caring for themselves after hospital discharge.

The second group of participants consisted of hospital discharge personnel. The investigators contacted directors of the hospital discharge departments at several metro hospitals to request permission to send an electronic survey about the discharge of patients experiencing homelessness to their discharge personnel and social workers. Once the hospital's permission had been obtained, participants were emailed a link to the survey. The survey consisted of five questions from the National Health Care of the Homeless Council: (1) How often do you encounter patients who are experiencing homelessness? (2) What gender are the patients who are experiencing homelessness? (3) How often is the discharge delayed due to homelessness? 4) Where are patients experiencing homelessness typically referred to at discharge? and (5) What do you perceive as the biggest gaps in the community related to homelessness?^1^ The participants returned the survey through the electronic system upon completion.

**Table:** Health questions, group 1 (N = 20)

Symptom	Number of participants (%)	Participant comments
Difficulty walking	17 (85%)	unsteady gait lower limb and toe amputations diabetic neuropathy degenerative joint disease knee pain instability, open foot wound, stroke
Poor vision	18 (90%)	corrective lenses cataracts glaucoma eye injury retinopathy poor night vision
Fatigue/exhaustion	15 (75%)	shortness of breath heart failure COPD heavy work assignment night shift difficulty day sleeping chronic back pain blood glucose level fluctuations
Daily pain	19 (95%)	musculoskeletal pain neuropathic pain headaches phantom pain
Falls in the past 3 months	13 (65%)	shortness of breath from heart failure weakness hemiparesis from stroke tripping over feet falling over furniture falling in the bathroom falling due to meds falling on ice falling during a seizure daily falls or falls several times each month
Inability to independently perform ADL	4 (20%)	dressing carrying food trays laundry showering navigating stairs toileting personal hygiene
Anxiety/depression	9 (45%)	anxiety/nervousness inability to control worry depression hopelessness
Mental/behavioral health issues	18 (90%)	chronic depression post-traumatic stress disorder paranoia, bipolar disorder attention-deficit/hyperactivity disorder nightmares
Dentition issues	15 (75%)	edentulism broken teeth tooth extractions cavities inherited bad teeth methamphetamine dental disease limited access dental care access

The third group consisted of homeless shelter staff interviewed by the investigators. The homeless shelter directors in the midwestern community were participants in the coalition and were asked electronic email by the coalition directors to participate in the study. The researchers followed up on any who volunteered to be interviewed. The homeless shelter directors in the midwestern community were asked through electronic mail from the coalition directors to participate in the study and were interviewed about the experience of receiving guests from healthcare facilities. The interviews were recorded with permission and transcribed verbatim. Participants were allowed to complete the questions via an electronic survey link if preferred for convenience. The survey consisted of five questions from the National Health Care for the Homeless Council: (1) Over the past six months, have shelter guests come to your facility with health illnesses and injuries? (2) How many guests had just received medical care? (3) Did the individuals come with the necessary medications and medical supplies that were needed when they were admitted? (4) Were the individuals independent in their ADL? and (5) How much time do you think individuals might have benefited from a medical respite program had there been one in the community?^1^

## Results

### Difficulties posthospitalization

Twenty adults experiencing homelessness were interviewed. They reported 25 hospitalizations and over 50 ED visits in the year before the study. The mean age of these participants was 40 years old. The majority were male, non-Hispanic White, had a high school education, and had been homeless on multiple occasions (see *Demographic results, group 1*).

Participants reported being hospitalized for health conditions including heart failure, lung and kidney cancer, hypertension, chronic obstructive pulmonary disease, diabetes, wound infections, COVID-19 pneumonia, complications from HIV infection, bipolar disorder, posttraumatic stress disorder, depression, anxiety, paranoia, and substance use disorders. Most participants reported experiencing difficulty walking, poor vision, physical fatigue and exhaustion, daily pain, falls within the past 3 months, mental health and behavioral issues, and dental issues. Reports of anxiety and depression (45%) represented many symptoms of those experiencing homelessness (see *Health questions, group 1*).

Participants were also asked about the difficulties and support they had in caring for themselves after being discharged. They described the inability to remember and follow discharge instructions; an inability to perform dressing changes using a clean technique; a lack of support from others; and a lack of supplies, equipment, and/or medications. They often returned to the ED when they experienced a further exacerbation of symptoms. Several participants described the social support they received (see *Participant descriptions of social support*).

### Discharge difficulties

Twenty discharge planners from three hospitals participated in the electronic survey. Fifteen participants (75%) reported encountering patients experiencing homelessness one or more times each week. Half (50%) of the patients identified as male, and the other half (50%) as female.

Participants reported that hospitals have difficulty discharging patients who are homeless if there are any ongoing health needs or a need for follow-up services due to the lack of health insurance or inability to pay. Discharge is often delayed, and the hospital stay is prolonged when individuals could not be admitted to skilled or long-term-care facilities as needed. If there is a delay in discharge, the hospital bed is not available for another ill patient. Discharge planners reported community service gaps as a lack of shelter beds and shelters capable of caring for patients with medical needs, skilled care facilities, transportation, and mental health resources.

### Difficulties for shelters

Five shelter staff from three residential homeless shelters volunteered to participate in the study. Shelter staff reported that guests have arrived at shelters following hospitalization without notice and that admission to the shelter may not always be appropriate for the guests' needs. For instance, guests lacked medications and durable medical equipment. They were not always able to perform ADL, and the shelters generally did not have trained staff to meet these needs. Furthermore, palliative, hospice, and long-term care beds were sometimes needed for guests and were not available. In addition, shelter staff reported that guests often needed mental health and substance use services, which they could not provide.

Shelter staff reported that 1 to 3 weeks of medical respite before admission to the shelter would likely be adequate to regain strength and increase their ability to perform ADL.

## Discussion

The results of this study are consistent with the literature reporting that adults experiencing homelessness frequently experience disease processes and adverse symptoms and may use ED services to meet these needs due to lack of insurance and inability to pay for healthcare services.^4^ Because individuals are not receiving consistent care in a primary care setting, diseases may exacerbate until hospitalization is required. Continued recovery time is often needed after a hospital stay and the individual experiencing homelessness may lack resources and assistance with self-care. Discharging to a safe, appropriate place where further follow-up and recovery can occur may be difficult for discharge planners to accomplish. Residential homeless shelters are often not equipped or staffed to care for individuals with complex health needs.

Four of the themes identified by Canham et al.^10^ are especially noteworthy regarding the current study.^10^ “Communication/coordination” described a lack of coordination between hospitals and housing services which impacted the anxiety and recovery of the persons experiencing homelessness. The authors also reported that shelters sometimes received discharged patients unexpectedly arriving at the shelter without advanced notification, so shelters could not provide appropriately for the individual's needs. In the current study, the shelter staff communicated with providers, social services, and skilled nursing facilities to find the most satisfactory location for the individual to meet their needs. This was beyond the scope of the role of shelter personnel.

“Supports for After-Care” was described as identifying a need for immediate and long-term support for individuals with medically complex needs.^10^ In the current study, shelter staff reported a lack of available services such as mental health services, addiction treatment, supportive housing, and resources for the individual beyond immediate needs for medications and medical equipment.

*“Complex Medical Care and Medication Management”* described the lack of instruction available for shelters to assist guests recently discharged from a hospital, the lack of medications and medical equipment, and the lack of staff qualified to provide this care.^10^ In the current study, shelter staff reported a lack of nursing personnel and equipment to perform dressing changes, monitor blood glucose levels, and ensure an adequate supply of medications was available for guests.

“Basic Needs and Transportation” described the needs of clothing, food, money, housing, and transportation to assist the person experiencing homelessness with recovery.^10^ Transport was especially needed to get individuals to their destination due to weakness from the hospital stay and the necessity of follow-up appointments.^10^ Consistent with Canham et al.,^10^ discharge planners and shelter staff in the current study reported that shelters had similar difficulties in providing appropriate placements, transport, and assistance in completing applications for additional resources.

## CONCLUSION

Individuals experiencing homelessness may have health concerns that often go untreated or undertreated, resulting in poor health outcomes. This study contributes to a better understanding of the healthcare service gaps for individuals experiencing homelessness when medical respite care is unavailable. These perspectives also inform nurses of the complexity of the problem so they may better advocate for these patients and assist with necessary care transitions.

## Participant descriptions of social support

“I did okay because I ended up going back to the shelter. If I didn't go there, then I would not have had none of it. I would have ended up not having my medicine or anything.”

“When I'm not here (in the shelter) or places like this, I kinda don't really care about myself. Especially on my foot ‘cause I was here last year, and I was taking good care of it here, but once I left, I stopped taking care of it because I didn't have the supplies I needed and also because I kinda just didn't want to. And then it got a lot worse. As soon as I got back here, I started taking care of it again and it just got better. But being here we got a bed, and we're around people. It's just more of a want to do it because I'm here.”
